# Identification and Assessment of the Driving Forces behind Changes in the Foothill Landscape: Case Studies of the Mysłakowice and Jelenia Góra Communities in Poland

**DOI:** 10.3390/ijerph191610462

**Published:** 2022-08-22

**Authors:** Piotr Krajewski, Monika Lebiedzińska, Iga Kołodyńska

**Affiliations:** Institute of Spatial Management, Wrocław University of Environmental and Life Sciences, 50-375 Wrocław, Poland

**Keywords:** driving forces, landscape change, landscape change index, land-use change, drivers

## Abstract

The main objective of this study was to determine the driving forces behind landscape change and the perceptions of change by the residents of selected research areas. The communities used for the study were Mysłakowice and Jelenia Góra, located in the Lower Silesia region in Poland. Mysłakowice is a rural community, and Jelenia Góra is an urban community. The landscape of both municipalities is dominated by forest-covered mountains surrounding dispersed built-up and agricultural areas. The time range of the analysis was 2005–2020, covering the period after Poland’s accession to the European Union, and was divided into the following three time periods: 2005–2010, 2010–2015, and 2015–2020. The research methodology consisted of the following three stages: (1) the identification of landscape changes on the basis of land cover data and the calculation of the landscape change index (LCI), (2) the characterization and classification of the identified landscape changes, and (3) the identification of the driving forces of landscape changes through surveys with the residents of both municipalities. The results obtained based on the surveys were often consistent with the results from the GIS analysis. The respondents were able to identify the most important changes and proposed the driving forces affecting them. According to the residents of Mysłakowice and Jelenia Góra, the changes in the landscape between 2005 and 2020 were primarily the result of political and socio-economic driving forces, accompanied by forces from other groups. However, each time period was distinctive. The analysis showed which types of changes in the landscape were viewed positively and negatively by the people during the analyzed periods of time, and what the influence of the different driving forces was on the formation of changes in the landscape.

## 1. Introduction

In recent years, more and more attention has been paid to the analysis of the forces which, over time, have caused noticeable changes to the landscape, and which have significantly influenced the direction of further transformations of European landscapes, especially in Central Eastern Europe. In order to fully understand landscape transformations, the proximate and underlying causes and processes of change must be identified, which means identifying the driving forces [1]. These are also called drivers [2] or key processes [3]. Driving forces are all processes that affect landscape change and can be identified over time. They constitute a complex system of interactions that together cause the initiation of specific changes in the environment [4]. Many different forces can act on each change simultaneously and what is identified as the driving force is primarily dependent on spatial and temporal scales. Forces vary in origin, scope and scale, duration, intensity, and nature. The authors distinguish several divisions of forces according to the mentioned differences, but emphasize the fact that this is not a closed catalog [5]. Bürgi uses five main types of driving forces in his work, which are socio-economic forces, political forces, technological forces, natural forces and cultural forces [4]. Another division of driving forces distinguishes between direct and indirect driving forces [5]. Generalization is partly needed because it is impossible to interpret all the drivers of change [4].

The importance of GIS systems is emphasized in the study of driving forces. Most researchers use ArcGIS [4,5,6]. Geographic information systems provide a number of opportunities to perform spatial analyses based on spatial data [7,8,9]. In order to understand the driving forces, it is worthwhile to explore the links between the natural environment and people, not only because of possible changes in society, but also because of the increasing importance of participatory methods in analyses. Analyses of landscape change can be extended to include human-observed changes [4]. Frequently during interviews, a participant can help explain changes that are difficult for an outsider to identify due to the outsider having less knowledge of the details of the area [10]. Researchers use face-to-face interviews [1,11], surveys [12,13], group interviews, focus groups, and field observations [14] to learn about opinions. In this study, a survey was conducted to find out the perceptions of the residents of the communities under study. Residents were given the opportunity to provide information about their feelings towards changes in the landscape. The changes may have significantly improved or worsened their existing living conditions [1].

Research on driving forces is divided into three types, including local or regional case studies, larger-scale multi-country studies, and comparative studies of past research. At the local level, site-specific landscape changes are identified, while larger-scale studies are designed to identify major trends in change. Meta-analyses provide some kind of benchmarks and opportunities to identify driving forces for future case studies [15]. This article is a case study at the local level.

Research on driving forces has been steadily growing and gaining interest. There is an increasing trend in the number of publications; in 1995–1999, there were 10 studies, in 2000–2004, there were 15 studies, and in 2005–2015, there was a clear jump in the analysis of driving forces, with 117 studies conducted. Articles analyzing only one area and having one spatial scale were clearly dominant. The temporal periods of their research were separated for analysis and were evenly distributed into two, as well as three or four, compartments. Among the indirect driving forces, political factors, in the form of agricultural, forestry, nature conservation, and climate policies, or spatial development, were most often cited as having a direct impact on the other indirect driving forces. This was followed by natural or spatial driving forces and cultural forces [15].

The growing number of subsidies and other measures associated with accession to the European Union have influenced the overall development of the countries. There have been changes in land use, road network development, and water and sewage infrastructure. Cities began to grow, villages near urban centers became peripheral areas, the accessibility of remote places to cities increased, and technology continued to develop. Technological changes were mainly related to mechanization in agriculture or industrial development. Culturally, entry into the European Union involved an attempt to change life preferences to those found on the western side of the continent and an increasing environmental awareness. Facilitated opportunities to travel in European countries, the lesser importance of permanent place of residence, a higher standard of living in the West and learning from other cultures, as well as educating the public on the need to protect the common European heritage and access to European funds, have led to positive increased attention to the needs of leisure, self-realization and the condition of the surrounding space. All this influenced a significant number of changes in the landscape [16]. Such a situation occurred in Poland. Accession to the European Union in 2004 was a key driving force for subsequent changes. Research on driving forces in Poland is still not extensive. We can distinguish the studies of Ślężański Park Krajobrazowy for the period of 1883–2013 [17], the mountain areas of the Sudety Mountains for the period of 1746–2000 [18], the landscape parks located in Dolnośląskie Province for the period of 2000–2018 [19], the mining areas of Belchatów and Turoszów for the years 1940–2011 [20], the rural communes of the Upper Silesian and Zagłębie metropolitan area for the years 2000–2018 [21], as well as the communities of Kąty Wrocławskie and Ostrów Wielkopolski [22]. There are many more studies in other countries of Europe. The analyses of driving forces has mainly concerned the landscapes in Switzerland [1], Germany [7], the Czech Republic [23], Slovakia [24], or Mediterranean landscapes [12]. Some studies compare case studies from different parts of Europe [25]. Urbanization, particularly in suburban areas, has been highlighted as the main cause of many changes [26], resulting in an increased percentage of urbanized landscapes and fewer areas of semi-natural landscapes. Other reasons for landscape changes are agricultural intensification, plant succession, increased demand for service areas, the development of renewable energy sources, and the enlargement of protected areas [27].

The main aim of our studies was to identify the driving forces of landscape change in the period of 2005–2020 and to determine which forces have had the greatest influence on landscape changes. We used quantitative tools for identifying the types of landscape transformations over time and qualitative tools for capturing citizens’ perceptions of changes. We integrated spatial data from analyzing archival orthophoto maps with social perception studies (online surveys). Our objectives were as follows: (1) to quantify and classify landscape changes in two case studies representing very popular mountainous urban and rural landscapes, (2) to identify underlying drivers behind landscape changes using a social research method, and (3) to assess what kind of forces were crucial in creating landscape changes. This article seeks to answer the three formulated research questions, as follows:

I. What is the level of landscape change in the analyzed communities?
II. What are the dominant driving forces in each period of time in the analyzed communities?
III. Are the changes at the same level, or do they depend on the time period or area in which they occurred?

## 2. Materials and Methods

### 2.1. Case Study Area

**Figure 1:**
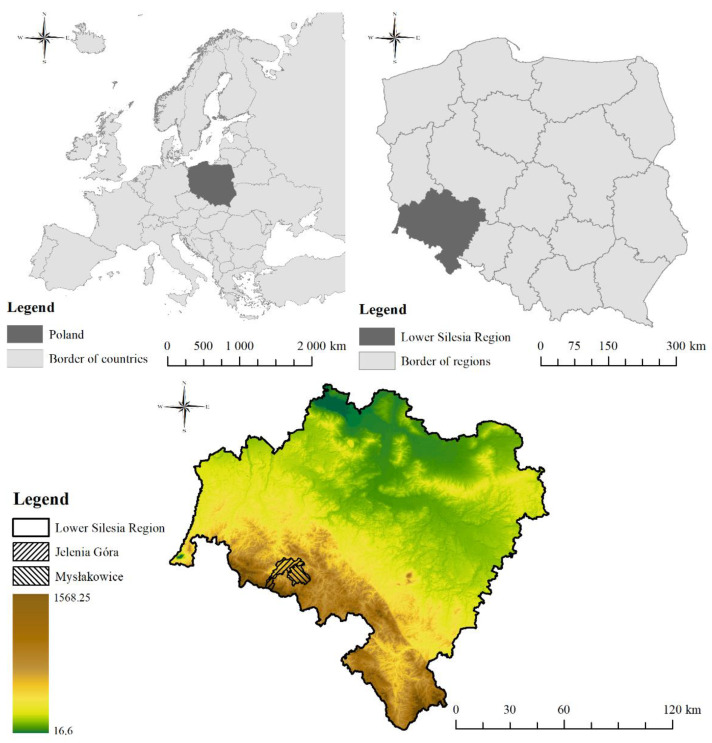
Location of the communities on the background of Europe, Poland, and the Lower Silesia region.

The research area comprises two communities located in the southern part of Poland and the Lower Silesia region (Figure 1). The Mysłakowice commune and the city of Jelenia Góra are located in a very picturesque, mountainous area.

The area of the Mysłakowice commune is 87.96 km^2^, and it is located at an altitude of 343.63 to 933.11 m. The Mysłakowice commune is almost entirely located in the Jeleniogórska Valley mesoregion, and the eastern side of the commune is located in the Rudawy Janowickie mesoregion. Almost half of the land is composed of all kinds of agricultural areas, and a slightly smaller part of the land is made up of forested areas. The population of the commune as of 2020 was 10,104 people. The community’s population mostly had an upward trend between 2005 and 2020. Within the borders of the commune, there is the Rudawski Landscape Park, the Natura 2000 Area Karpnickie Ponds, and palace and park complexes belonging to the Valley of Castles and Gardens. The main road in the Mysłakowice commune is Provincial Road no. 367, connecting Jelenia Góra and Wałbrzych. There is one active railroad station serving passenger traffic in Wojanów on the Wroclaw–Szklarska Poreba route. The development of the Mysłakowice commune is based mostly on tourism and recreation, combined with agriculture.

Jelenia Góra has an area of 109.29 km^2^ and is situated at an altitude of 318.71 to 1420.7 m. The town of Jelenia Góra is located mostly in the Jeleniogórska Valley mesoregion; however, the south part of town is located in the Karkonosze mesoregion, and the northwest part of town is located in the Izerskie Plateau mesoregion. Jelenia Góra is characterized by numerous forms of natural protection. The strictest form of natural protection is the Karkonosze National Park with its buffer zone in the southern part of the city. There are also four Natura 2000 areas within the borders of the city. In terms of landscape values, the view of the mountain massifs surrounding the Jelenia Góra Valley in the form of the Karkonosze and a fragment of the Izerskie Mountains are particularly outstanding. The population of the city of Jelenia Góra is gradually falling, as in other former provincial towns. The number of inhabitants in 2005 was 87,017 people, and it was 78,335 people in 2020. Communication is very well developed. One of the most important roads is the National Road no. 3, which leads to the Baltic Sea. Railroad transport includes five stations in different parts of the town on the route connecting Wrocław and Szklarska Poręba. The town is developing economically. The landscape values are also conducive to the development of tourism.

### 2.2. Identification of Landscape Changes

#### 2.2.1. Research Procedure and Data

The landscape changes in the communities of Mysłakowice and Jelenia Góra during the three time periods of 2005–2010, 2010–2015, and 2015–2020 were analyzed. The first stage of the study was to classify land cover types (Table 1). A total of 15 classes were divided into the following 3 groups:

(A) Cultural landscape elements;
(B) Cultural and natural landscape elements;
(C) Natural landscape elements.

**Table 1:** Classification of land cover types.

Category of the Landscape Elements	Landscape Elements	Code of the Landscape Elements
Cultural landscape elements	Residential area	A1
	Roads and rail networks and associated land	A2
	Service and industry area	A3
	Ports and airports	A4
	Mining area, construction area	A5
Cultural and natural landscape elements	Parks, and sport and leisure area	B1
	Meadows and pastures	B2
	Arable land	B3
	Orchards, vineyards, and plantations	B4
	Other non-categorized areas	B5
Natural landscape elements	Forest area	C1
	Scrub and/or herbaceous vegetation associations	C2
	Bare land (areas with little vegetation)	C3
	Wetland	C4
	Water area	C5

According to the definition of landscape from the European Landscape Convention, a landscape is “an area perceived by people whose character is the result of the action and interaction of natural and/or human factors”. In the adopted classification, the cultural (anthropogenic) elements of the landscape included those whose creation was primarily contributed by humans, the cultural–natural elements included those elements that are the interaction of anthropogenic and natural elements, and the group of natural elements includes those for the formation of which natural forces are primarily responsible. Then, archival orthophotos for the years 2005, 2010, 2015, and 2020 obtained from the Central Office of Cartography and Geodesy in Poland were collected. The raster data formed the basis for the vectorization of land cover types in ArcGIS software. The resulting data on the total area of land cover types enabled the calculation of the landscape change index (LCI) developed by Krajewski et al. [28]. The index illustrates the level of change that occurred in the landscape during each period of time. Vector data for each period were intersected with each other in ArcGIS. This resulted in data on specific changes from one land cover type to another. Based on the identified changes, the processes of change occurring in the landscape were determined. A diagnostic survey was then conducted among the local population to determine the driving forces affecting change.

#### 2.2.2. Identification of the Level of Landscape Changes

The value of the landscape change index numerically informs us about the degree of the intensity of changes, but does not provide information about the typology of the transformations that occurred [17,19].

In order to calculate the index of landscape change, area data for each land cover type were needed for all the years studied. The area data were used to calculate CAi values for each land cover type using the following formula:

CAi = ((At + 1) − At)/TA × 100(1)

where CAi is the difference in the percentage of a given land cover type; At + 1 is the area of a given land cover type from a later year; At is the area of a given land cover type from an earlier year; and TA is the area of the study area.

Multiplying the resulting value times 100 yielded the percentages. Here, CAi can be either a positive or negative number depending on whether the land cover type increased or decreased in the subsequent year under analysis. Both positive and negative values represent changes in the landscape that have occurred. For this reason, absolute values from the results obtained are used to calculate the landscape variability index. The LCI is calculated using the following formula:

(2) $$\mathrm{LCI}\;=\overset{n}{\underset{i=1}{\sum }}\mathrm{CAi}$$

where LCI is a landscape change index, and CAi is a difference in the percentage of a given land cover.

#### 2.2.3. Identification of the Character of Landscape Changes

The next stage of the study was the classification of landscape transformation types within the study areas. We identified 12 processes which determine the type of transformation, as follows:

(1) Urbanization—changes from other land cover types to residential areas;
(2) Industrialization—changes from other land cover types to industrial and commercial areas;
(3) Development of transportation areas—changes from other land cover types to road and railroad land;
(4) Development of recreational areas—the creation of parks, sport buildings and leisure areas on built-up areas or agricultural areas;
(5) Intensification of agriculture—changes from meadows and pastures to arable land and changes from previous areas not used for agriculture to arable land, meadows, pastures, plantations, and orchards;
(6) Extensification of agriculture—changes from arable land to meadows and pastures;
(7) Set-aside land—changes from arable land to other land and areas with little vegetation;
(8) Afforestation—changes from agricultural land and areas with little vegetation to forest land;
(9) Deforestation—changes from forest land to other land cover types, in particular arable land, meadows, and pastures, and communities with little vegetation;
(10) Natural succession—changes to woody and shrubby vegetation communities from meadows and pastures, arable land, plantations, or areas with little vegetation, as well as changes from areas associated with communication to areas with little vegetation;
(11) Water resource management—changes from anthropogenic, agricultural, and forest areas to water areas and vice-versa;
(12) Wetlands—changes from forest, arable land, and building areas to wetlands.

Each of the changes was assigned to a process. This provided data on the total area where the process took place.

#### 2.2.4. Identification of Driving Forces by an Online Survey

Driving forces were identified using an online survey filled in between February and April 2022. We used voluntary response sampling (a type of non-probability sampling) for the online survey that was posted in discussion groups on social media that used the name of the community. The survey included individuals who, as volunteers, were willing to complete the survey of their own choice. We cannot estimate the size of the population that could have participated in the survey; that is why the sample size in not a representative sample. The survey consisted of three main sections. One of the sections included background information, such as age, the period of time in which they have lived in the community, education, social status, and gender. The second section included questions concerning the following topics:

(1) The time period with the greatest changes;
(2) A list of the three most important changes in their opinion;
(3) An evaluation of the changes;
(4) An identification of the most frequent types of transformations;
(5) The specification of the areas which are most frequently subject to changes;
(6) The identification of the land cover types that are more and more abundant.

The third section presented the most specific areas of change in the analyzed time periods and asked the respondents to tick off an unlimited amount of driving forces that they believe may have influenced the creation of such a change in the landscape from the following five groups of forces: socio-economic, political, technological, natural/spatial, and cultural, according to the classification proposed by Bürgi et al. [4]. Particular driving forces were assigned to the mentioned categories according to the classification of the most frequently analyzed forces indicated by Plieninger et al. [15]. However, in order not to make the question difficult for respondents to understand, the questionnaire did not use a categorization of driving forces, but rather listed them all.

The questionnaires included both closed-ended questions, forcing respondents to make judgments on an adopted scale, as well as allowing them to add their own thoughts and opinions on questions about the most important changes in the landscape and their driving forces. The final element of the research was a qualitative analysis of the indications and opinions obtained. This allowed for a more complete understanding of the negative and positive changes that occurred during the surveyed periods, and the completion of the catalog of driving forces that were not included in the indicated list. The full questionnaire for the municipality of Jelenia Góra is in Supplementary Materials File S1, and the questionnaire for the municipality of Mysłakowice is presented in Supplementary Materials File S2.

## 3. Results

### 3.1. Identification of the Main Landscape Changes

In the Mysłakowice commune, the values of the landscape change index were similar in each period, especially in the second (1.73) and third period (1.80). The lowest value of the index occurred in the first period in Jelenia Góra (0.78). In Jelenia Góra, during the study periods of 2010–2015 and 2015–2020, the indicator reached the same value (1.74). The index values in the 2010–2015 period were very similar for each of the communities. The other two periods are characterized by greater variation (Table 2).

In the Mysłakowice commune, changes in the percentage of land cover occurred in 12 land cover types. The highest decreases (−1.61%) were observed for meadows and pastures in the period of 2010–2015 and were slightly smaller for arable land (−1.20%) in the period of 2005–2010. The highest increase in the proportion of land cover forms was for meadows and pastures in the 2015–2020 period (+1.04%) and scrub and/or herbaceous vegetation associations in the 2010–2015 period (+0.74%). There is a noticeable decrease in residential areas for the most recent study period (Table 2).

In Jelenia Góra, changes in the percentage of land cover occurred in 13 land cover types. The highest decline occurred for meadows and pastures for the 2015–2020 period (−0.99%). High declines for each study period were observed for arable land. The highest decrease in arable land was in the 2015–2020 period (−0.74%). Land cover percentages increased the most in the 2015–2020 period for roads and rail networks and associated land (+0.72%). High increases are also observed for forest areas (+0.49%) and scrub and/or herbaceous vegetation associations (+0.45%) during the 2010–2015 period. Increases in residential areas were relatively small compared to other land cover types, with a decrease observed during the first study period (Table 2).

### 3.2. Identification of the Character of Landscape Changes

At the second stage of the analysis, specific landscape changes were determined for the three analyzed periods.

The processes occurring in the landscape of the Mysłakowice commune are mainly related to changes in the types of crops and the emergence of new buildings. The dominant attractor in the commune is its landscape values. Locations within picturesque mountain areas, along with palace–park complexes, lead to the development of settlements, among other things. Unfortunately, the commune is characterized by a small coverage of spatial development plans. In 2010, it was 7.6% of the commune’s area; in 2015, it increased to 30.1%; by the end of 2020, it had not increased. Instead, about 50 planning permits are issued annually in the community.

**Figure 2:**
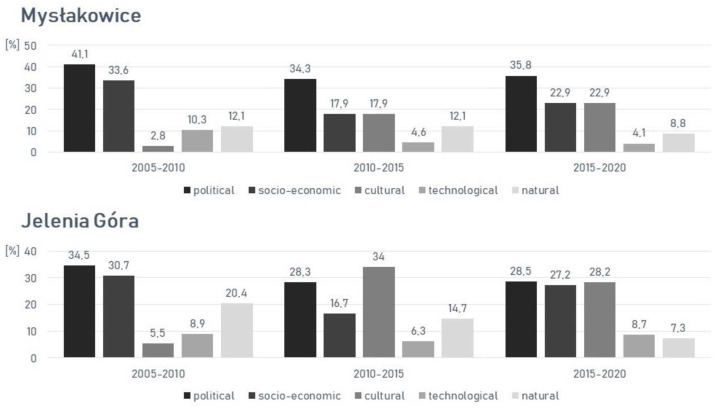
Five main types of driving forces in the communities.

In the Mysłakowice commune, 83.33 ha of land area changed in the period of 2005–2010. Most changes occurred on the western side of the commune, while almost none occurred in the eastern part (Figure 2). This is due to the presence of mountains and a large area of forest in the eastern part of the municipality. In the period of 2005–2010, in the largest area of the Mysłakowice commune (24.07 ha), there have been processes of change determined by the intensification of agriculture. These changes may have resulted from the possibility of receiving subsidies per hectare from the EU funds, especially for organic farming, so it was decided to increase cultivation. Similarly, it was assumed that EU plans had an effect on the maintenance of natural meadows and pastures, which also increased in the commune at that time. The second distinctive process was urbanization (23.25 ha). Previous agricultural areas were transformed into residential areas, resulting in the fragmentation of the landscape. The process referred to as water resource management was also visible (17.07 ha). As a result of beaver activity, wetland occurred over an area of 2.18 ha (Table 3), which was also a frequent phenomenon. The first period was additionally characterized by the lack of changes within forest areas, which proves the stability of these areas.

**Figure 3:**
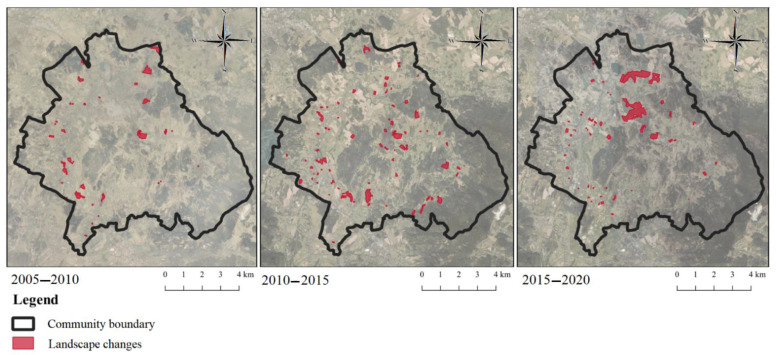
Location of the landscape changes in the Mysłakowice commune in 2005–2010, 2010–2015 and, 2015–2020, respectively.

In the period of 2010–2015 in the commune of Mysłakowice, more changes occurred compared to the previous period. The total area of changes in the commune in this period was 139.15 ha. The changes occurred in the area of almost the entire commune, except for the eastern part (Figure 3). The years 2010–2015 in the Mysłakowice commune were characterized primarily by processes associated with the emergence of a larger area of scrub and/or herbaceous vegetation associations on agricultural land. The main process in this case was natural succession (38.71 ha). The process of urbanization took place on a larger area than in the previous period. New buildings were built on an area of 36.26 ha (Table 3). A process defined by water resource management occurred on an area of 24.66 ha. The least frequent processes in the second period were industrialization (0.82 ha) and deforestation (0.63 ha).

During the period of 2015–2020, land cover changes increased compared to the previous period. The total area of changes during this period was 183.94 ha. The changes were cumulative, especially in the northern part of the commune (Figure 3). In the period of 2015–2020 in the Mysłakowice commune, the dominant process was the extensification of agriculture (132.72 ha). The largest area of land was changed to meadows and pastures; the former arable land was particularly affected. These changes may have been influenced by the intense rains in 2017, with the consequent prolonged droughts and the lack of winter snow cover in the following years, so that the soils suffered, and it was more beneficial to establish meadows and pastures. Increasing per hectare payments from year to year may also have been important. The second outstanding process in the commune in the last period was the management of water resources (21.18 ha). Some breeding ponds were temporarily deprived of water and some of them were permanently closed. The third process in terms of area change was urbanization. Changes associated with new development occurred on 17.82 ha.

In Jelenia Góra, the dominant processes of change were typically natural, and concerned forest and agricultural areas. The changes associated with the emergence of new buildings in Jelenia Gora were not the most significant. This can be associated with a constant decrease in the population in the city. The development of buildings in the first two periods was observable in the Jagniątków district located in the Karkonosze part of the city. Buildings in this part were mostly boarding houses. The reason for those changes was the pressure connected with the development of tourism. The dominant attractor in Jelenia Góra is the presence of Karkonosze and the distinctive landscape values.

**Figure 4:**
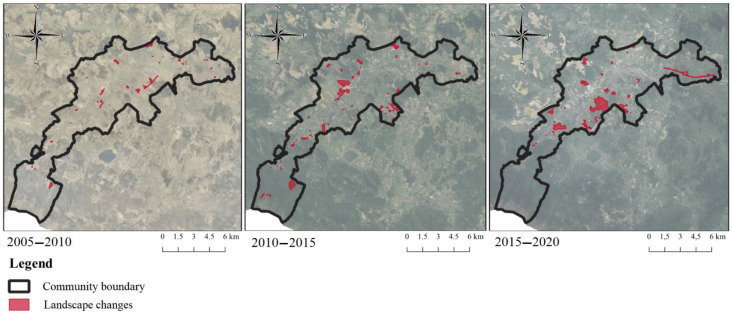
Location of the landscape changes in the city of Jelenia Góra in 2005–2010, 2010–2015 and 2015–2020, respectively.

In the years 2005–2010, a total of 82.60 ha was changed in Jelenia Góra. The changes occurred in almost the entire community, with the highest accumulation in the city center (Figure 4). In the years 2005–2010, the process of the extensification of agriculture stands out in particular (24.02 ha). However, it is worth noting that meadows and pastures were also very susceptible to other processes in the landscape, such as changes resulting from the emergence of new buildings, or natural succession in the area. Natural succession was another outstanding process (16.85 ha). It was often characterized by certain areas changing to tree and shrub vegetation communities. In Jelenia Góra, the process of deforestation occurred in the area of Karkonosze (9.03 ha). This type of change could have been influenced by climate, soil properties, or even a natural disaster. In this period, 9.41 ha of the site underwent changes resulting from new buildings, especially in Maciejowa and by the newly constructed Provincial Road no. 367. The construction of the road was an attractor of changes in each period.

Changes between 2010 and 2015 in Jelenia Góra had a total area of 241.52 ha. There were significantly more changes compared to the previous period. The largest accumulation of changes occurred in the southeast, near the direct border with the commune of Mysłakowice (Figure 3). This is due to the intensive development of built-up areas, which is linked to lower land prices in suburban areas of Jelenia Góra. The period of changes in 2010–2015 was dominated by the extensification of agriculture (57.75 ha). The second prominent process was afforestation (57.59 ha). These changes often affected the Karkonosze area; thus, changes related to deforestation in the earlier period were corrected. A distinctive area (35.18 ha) underwent a process of deforestation. Tree and shrub vegetation communities were often changed to meadow and pasture areas. Another process that stood out between 2010 and 2015 was industrialization. Changes associated with the creation of new commercial and industrial areas occurred on 30.48 ha. Prominent commercial facilities that were built included the New Market Mall and the Sudecka Mall, which opened in 2015, and the Leroy Merlin built in the same period, but which opened in the next time frame. Of the industrial facilities, Jelenia Piast Sp. z o. o. or Zorka Sp. z o. o. in the city’s developing industrial zone stand out.

In the years 2015–2020, a total of 355.86 ha was changed in Jelenia Góra. The changes increased compared to the previous period. The highest density of changes occurred in the south-eastern part of the city (Figure 4). The years 2015–2020, similar to the previous periods, were characterized by the dominance of the extensification of agriculture (250.59 ha). The mentioned process dominated all other processes that occurred in 2015–2020 in terms of area. The second most frequent process in the third period was the intensification of agriculture (47.62 ha). The third prominent process was urbanization (25.56 ha). New residential areas were particularly visible in the Czarne neighborhood and along Provincial Road number 367, which continued to be an attractor for further transformations, as in the first period. Transport areas were created across a comparable area (23.10 ha), mainly due to the Maciejowa bypass in the eastern part of the city.

### 3.3. Identification of the Driving Forces

#### 3.3.1. The Impact of the Changes on the Perception of the Landscape

The survey on landscape change involved 76 respondents from both communities. The demographic characteristics are presented in Table 4.

Among the 32 respondents from the Mysłakowice commune, the majority of the respondents (56.3%) lived in the commune for more than 20 years, while 31.3% lived there between 16 and 20 years, 9.4% lived there between 11 and 15 years, and only 3.1% lived in the commune between 5 and 10 years. The survey participants ranged in age from 26 to 62 years old. In the commune of Mysłakowice, 68.8% of the respondents described the level of change as high, and 28.1% described it as medium. Only 3.1% considered the changes to be low.

#### 3.3.2. Dominant Driving Forces Affecting Landscape Change

The residents of the Mysłakowice commune were presented with examples of changes in the landscape. The examples were characteristic for the analyzed years and could be recognized by the respondents. The questionnaires asked about agricultural intensification, natural succession, deforestation, water management, industrialization, and urbanization.

**Figure 5:**
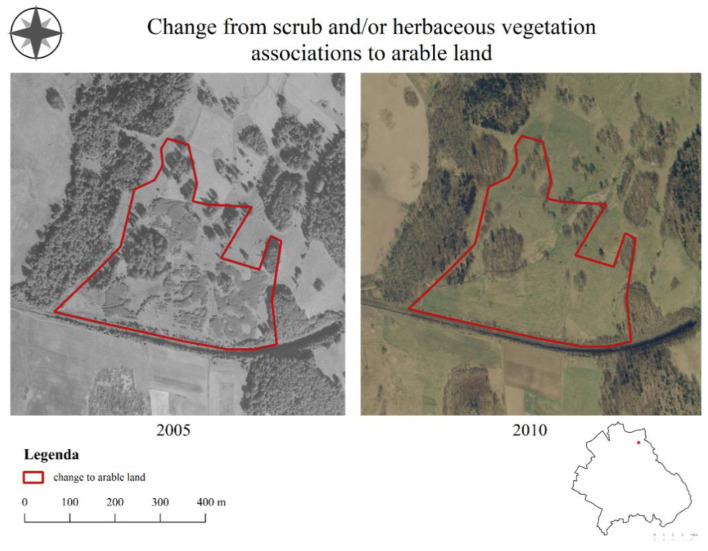
Intensification of the agriculture in the Mysłakowice commune.

According to the respondents, the intensification of agriculture (Figure 5) was most strongly influenced by the agricultural policy (75% of the respondents), by appointments in the structure of agriculture (62.5%) and by the properties of the soil (40.6%). The change was perceived by respondents as negative (25% very negative, and 28.1% negative) or neutral (40.6%).

Natural succession was most strongly affected by changes in the agricultural structure (65.6% of respondents), soil characteristics (56.3%), and agricultural and forestry policies (50%). The change was perceived positively (53.1% positive, and 31.3% very positive).

Afforestation was influenced by changes in the forestry structure (78.1% of respondents), forestry policies (75%), and nature conservation policies (40.6%). Afforestation was perceived positively (56.3% very positive, and 34.4% positive).

Water resource management was influenced by indirect forces, such as nature conservation and climate policy (50%), soil properties (40.6%), and the climate (37.5%). and direct forces, such as the construction of new water bodies (78.1%). Afforestation was perceived very positively (50% very positive, and 31.3% positive).

Industrialization was most affected by commercialization (90.6% of the respondents), property rights (81.3%), and the real estate market (59.4%). The change was perceived negatively by respondents (46.9% negative, and 15.6% very negative).

**Figure 6:**
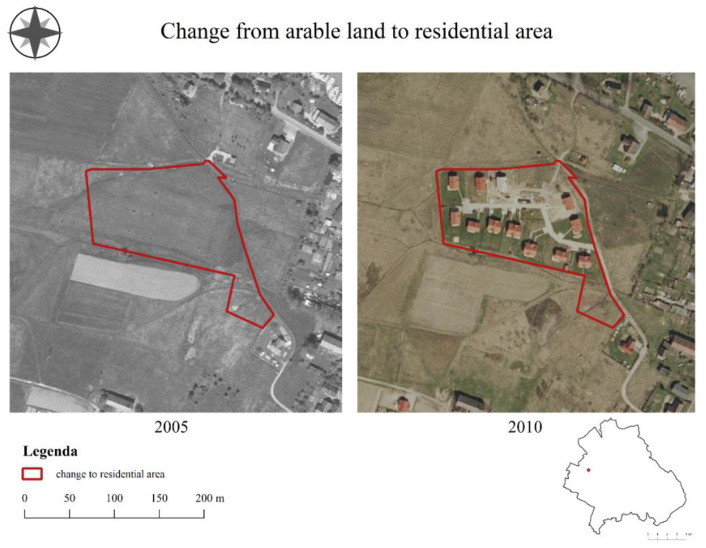
Urbanization in the Mysłakowice commune.

Urbanization in the form of compact development (Figure 6) was influenced by the forces of the real estate market (93.8% of respondents), property rights (87.5%), and the population and its distribution (87.5%). Opinions on this topic were divided (37.5% negative, 28.1% neutral, 25% positive, and 9.4% very negative). On the other hand, according to the residents of the commune, urbanization in the form of development as a result of zoning decisions was influenced by forces, such as property rights (96.9%), the real estate market (84.4%), and the population and its distribution (78.1%). These changes were perceived as negative (59.4% very negative, and 34.4% negative).

Residents of the city of Jelenia Gora were also asked about the characteristic and noticeable changes they saw. The survey asked about deforestation, urbanization, industrialization, and the development of transportation areas.

Of the indirect forces, deforestation was most strongly influenced by forest policy (70.5% of respondents), soil properties (54.5%), and climate (47.7%), with direct forces being natural disasters (61.4%). The changes were perceived negatively by respondents (50% negative, and 47.7% very negative).

Urbanization in meadows and pastures was most strongly influenced by the size of the population and its distribution (88.6%), the real estate market (81.8%), and property rights (65.9%). The changes were perceived positively by respondents (65.9% positive, and 11.4% very positive). In turn, urbanization in building areas was influenced by the real estate market (86.4%), the population and its distribution (81.8%), and property rights (70.5%). These changes were perceived even more positively (54.5% positive, and 36.4% very positive).

**Figure 7:**
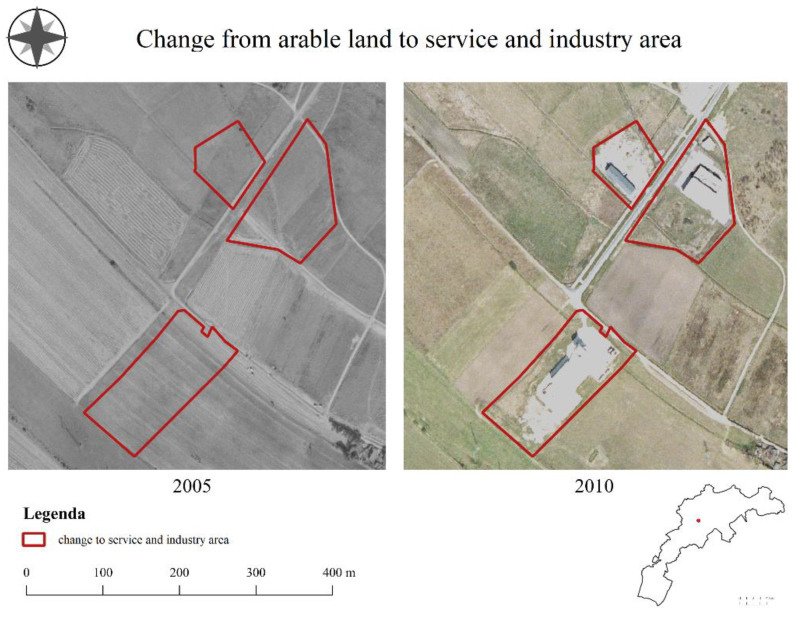
Development of transportation areas in the city of Jelenia Góra.

The industrialization process concerning retail facilities was influenced by forces, such as commercialization (81.8%), property rights (68.2%), and spatial development policies (54.5%). These changes were perceived as neutral or negative (43.2% neutral, and 38.6% negative). The industrialization of this type was perceived positively by 18.2% of respondents. On the other hand, industrialization resulting in the creation of warehouse facilities (Figure 7) was influenced by forces, such as spatial development policies (75% of respondents), commercialization (68.2%), and the technological modernization of society (43.2%). According to respondents, these changes were neutral or negative (56.8% neutral, and 31.8% negative). Commercial facilities were perceived by residents more positively than industrial facilities.

**Figure 8:**
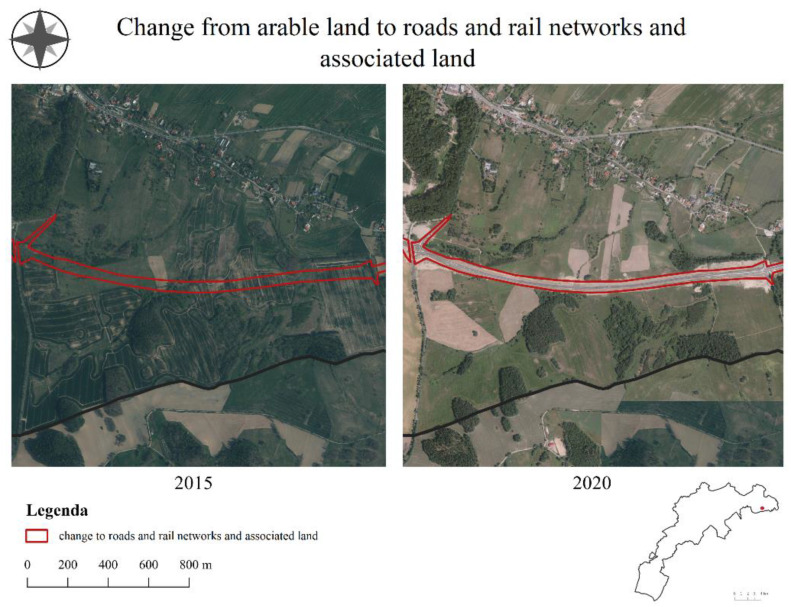
Development of transportation areas in the city of Jelenia Góra.

According to respondents, the development of transportation areas (Figure 8) was most influenced by the population and its distribution (88.6%), spatial development policies (68.2%), and topography (40.9%). These changes were perceived by respondents neutrally or positively (47.7% neutral, 29.5% positive, and 4.5% very positive).

In the Mysłakowice commune, respondents most often linked the changes to political driving forces. The first period had the highest share of political forces (41.1%), especially in the form of agricultural and forestry policies and property rights. This was related to the dominant process of agricultural intensification and urbanization in the 2005–2010 interval. The second dominant type of forces in each interval were socio-economic forces, especially changes in the structure of agriculture and the property market. The share of cultural forces differed the most across the three time periods analyzed. A greater share of cultural forces, particularly through population numbers and distribution in the 2010–2015 and 2015–2020 intervals, was associated with increased urbanization and industrialization (Figure 5 and Figure 6).

In the period of 2005–2010 in Jelenia Góra, respondents most often identified the impact of political (34.5%) and socio-economic (30.7%) forces. A high share of natural forces in both the first and second period was particularly associated with the processes of afforestation, deforestation, and natural succession in the Karkonosze part of the city. The increase in the share of cultural forces in the second period had to do with the increase in the area where urbanization, industrialization, and the development of transportation areas took place. The mentioned processes were strongly influenced by the population and its distribution. The interval 2015–2020 was characterized by the dominance of three types of driving forces, specifically political forces (28.5%), cultural forces (28.1%), and socio-economic forces (27.3%). The contribution of technological forces differed slightly in each of the periods studied. Technological forces exerted little influence on any change that occurred in the landscape (Figure 7).

#### 3.3.3. Qualitative Analysis of the Respondents’ Statements

The survey asked the inhabitants of the analyzed communities what changes were the most important in their opinion, and how they perceived them. Among the most important transformations in the community of Mysłakowice, respondents mentioned the dispersed development on agricultural land, which often did not relate to the current architectural style in the community. Respondents also drew attention to the smaller amount of greenery, more commercial and recreational facilities, and the renovation of culturally valuable buildings and areas. They mostly did not pay attention to changes within agricultural areas, such as the intensification or extensification of agriculture and natural succession, and it was these changes that dominated in the commune. A similar situation concerned the management of water resources. Residents were more likely to re-member anthropogenic changes that took place on agricultural land than more natural changes. This may be related to a particularly negative attitude toward urbanization in areas with significant landscape value. This is confirmed by the statements of the residents who have lived in the Mysłakowice commune for more than 20 years, as follows:

*“Increased number of newly built single-family houses in villages without spatial development plans, logging of forests, large number of billboards along roads obstructing landscapes”.*

(Respondent 1, age 40)

*“Changes are not beneficial. Increased number of inhabitants, private houses, cars, markets caused that the commune is no longer as picturesque as it used to be”.*

(Respondent 2, age 50)

*“Increased number of buildings in my opinion often deviating from the dominant style in the commune so this can be considered as negative changes. More recreational areas appeared at that time, a positive change of course. You can also see more care for palaces and parks, definitely a positive change”.*

(Respondent 3, age 44)

*“Houses and guest houses are being built in agricultural areas. There are more areas for sports and playing with children. The changes are positive”.*

(Respondent 4, age 41)

*“Less greenery and more houses everywhere”.*

(Respondent 5, age 52)

According to the respondents, the most important transformations in Jelenia Góra in the period of 2005–2020 were mostly discount stores, such as Gallery Sudecka and Gallery New Market, the construction of the Maciejowa Ring Road and Jana Pawła II Avenue, the construction of industrial buildings on Spółdzielcza Street, and the construction of the Termy Cieplickie aqua park. Respondents also drew attention to more bicycle paths, green areas, the renovation of buildings, the development of housing, and tourism. Most of the changes were described as positive. Most of the changes they described as negative were related to industrial facilities. This was confirmed by the statements of some residents who have lived in Jelenia Góra between 11 and 15 years, and those who have lived there more than 20 years, as follows:

*"Lots of new industrial areas, rather negative. More commercial areas, like malls, positive. Construction of national road number 3, positive”.*

(Respondent 1, age 51)

*“First of all the construction of national road number 3, partly positive because it reduced traffic near buildings and caused the development of tourism and the region, but it also represents more noise and pollution. New developments positive”.*

(Respondent 2, age 38)

*“The most noticeable was the construction of commercial facilities like the Sudeten Gallery, which was very much lacking before in such a large city. In terms of tourism development, the construction of thermal baths was also important. At present, the swimming pools, apart from the inhabitants, attract crowds from the whole voivodship and other places, so the change is very positive. In the nearest neighborhood there is also a noticeable new housing development. I perceived all this positively”.*

(Respondent 3, age 36)

*“The opening of the Termy Cieplice aqua park is a positive change and has a very good impact on the development and perception of the city. Discount stores on John Paul II Street—positive change through increased accessibility to goods and services. Bypass of Maciejowa positive change, road of better quality, influences tourism and affected the peace of Maciejowa inhabitants”.*

(Respondent 4, age 38)

## 4. Discussion

Landscape changes were most intense between 2015 and 2020. In the analyzed communes, landscape changes had an increasing trend. The changes identified by residents were characterized by high agreement with the results obtained from the analyses. However, not all transformation processes were noticed by the residents.

The classification of the types of transformation in both communities and the statements of the respondents made it possible to identify the main changes in the landscape. The main transformation processes in the Mysłakowice commune were the extensification of agriculture, natural succession, urbanization, and the management of water resources. In Jelenia Góra, the main processes were the extensification of agriculture, the intensification of agriculture, afforestation, natural succession, urbanization, industrialization, and the development of transportation areas.

In the commune of Mysłakowice, each period of time was characterized by a different process of change, with the intensification of agriculture in the first period, natural succession in the second period, and the extensification of agriculture in the third period. Urbanization and water resource management were also widespread. The residents of the Mysłakowice commune described urbanization as the most negative, rapid, and destructive of the scenic values. They perceived natural succession, afforestation, and the creation of new water reservoirs as positive. The dominant process in each interval in Jelenia Góra is the extensification of agriculture, but respondents did not notice it. For the Jelenia Góra residents, deforestation was the most negative change. They perceived industrialization mostly neutrally and less often negatively. Urbanization was perceived positively.

The residents of each community paid great attention to the development of recreational areas. These were not frequent changes, but they were very memorable to respondents. The vast majority of respondents did not remember changes in crop types, natural succession, or reforestation. Respondents particularly remembered changes that resulted in the construction of a residential, commercial, or industrial facility. More natural changes, which occurred gradually, were not clearly discernible or memorable. For this reason, natural changes were perceived positively by the respondents, while changes associated with the sudden construction of facilities were perceived rather negatively.

According to the residents of the communities, spatial development policies, other sectoral policies, and property rights combined with economic aspects had the greatest influence on the likelihood of change. The population of the community and how it was distributed was also very crucial. An increasing population will be associated with more residential, recreational, and industrial/commercial land in the community. In turn, the distribution of the population was associated with the development of communication areas, facilitating access to areas with a variety of functions. Less frequently, according to residents, human values and behaviors, or natural forces in the form of climate or topography, led to changes in the landscape. According to the respondents, the changes were mostly dictated from above, but the role of man is not excluded. In particular, in the case of the inhabitants of the Mysłakowice commune, urbanization was the result of human behavior.

Each commune was different, as was the period of time within each commune. The periods showed similarities in terms of the action of forces, but their share was never identical. The communes of Mysłakowice and Jelenia Góra were characterized by different areas, on which the identified transformation processes took place, but the greatest similarity between the periods was characterized by the level of urbanization. Mysłakowice, which is adjacent to the city of Jelenia Góra, is an attractive place to live in a rural area. This is a process observed in communities adjacent to cities.

The landscape change index determined the level of changes in the landscape for three time periods. The greatest differences in the level of changes were shown by the first period analyzed, while the greatest similarities were shown by the second period. Here, LCI values equal to 1 can be considered as normal dynamics of change within 5 years; larger and steadily increasing changes in subsequent years can be disturbing. The most dynamic changes in the landscape were made in Jelenia Góra. The scale of changes in Jelenia Góra in the first period was the smallest among all communities but, in the second period, it was the largest. The value of the landscape change index also reached the same value for the second and third interval. On the other hand, the rate of change in the commune of Mysłakowice was the most stable, but the scale of change in each period was ever-growing. The landscape variability index provides good information about the scale of changes in the landscape and can be used to draw general conclusions. However, it is worth conducting additional analyses that would show the scale of specific transformation processes.

In the commune of Mysłakowice, the changes may increase in the future due to the commune’s increasing activities related to the recent adoption of local plans and the desire of residents to settle in a quiet, peaceful commune with high landscape values. Residents did not like the existence of low plan coverage, and negatively perceived the housing developments resulting from decisions on land development conditions. Current activities in the commune have a chance to change their perception of landscape changes. Jelenia Góra, wanting to become a city with 100,000 inhabitants, has increased the number of residential areas within its borders but, as the data from the Central Statistical Office show, it is constantly depopulating. Additionally, the provisions of the spatial policy say that it wants to increase industrial and agricultural areas. As a result of these provisions, it can be expected that changes in the landscape in the coming years may take place across 2–3% of the area of the site.

## 5. Conclusions

This paper identifies the driving forces affecting landscape change in the communities of Mysłakowice and Jelenia Góra. The findings presented here are part of a research project that analyzed the driving forces behind changes in the landscapes of four other municipalities in the Lower Silesia region to include different locations and landscape types. This will avoid the local nature of the research and capture trends independent of location and landscape type. The study showed that changes were most intense in the 2015–2020 period and least intense in the 2005–2010 period. The area of changes in the communes was larger in each subsequent time period. A total of 12 types of transformations were identified in the Mysłakowice commune, and 11 types of transformations were identified in the city of Jelenia Góra. In both communities, residents noted residential and commercial/industrial development on agricultural land the most. However, the dominant underlying driving changes were usually the intensification or extensification of agriculture. These changes were less noticeable and were usually perceived neutrally. Changes associated with the development of residential buildings in the commune of Mysłakowice were perceived negatively, but positively in Jelenia Góra. The emergence of new industrial–service buildings in both communes was assessed negatively in relation to industrial buildings and neutrally or negatively in relation to service buildings. The development of transport areas was assessed as neutral or positive. Changes associated with natural succession were assessed positively, as were afforestation and the creation of new water bodies.

Residents of both study areas identified driving forces from the following five groups: political, socioeconomic, cultural, technological, and natural. Political forces combined with socio-economic forces were most likely to influence changes in the landscape in all of analyzed case studies. Spatial development policies affecting all kinds of landscape changes were most often cited. Landscape planning should take into account the views of residents. Changes should not negatively impact residents’ perceptions but should rather improve their existing quality of life.

## Figures and Tables

**Table 2:** The percentage of deviation for the types of land cover in the communities of Mysłakowice and Jelenia Góra in the analyzed periods.

Land Cover Classes	Mysłakowice			Jelenia Góra		
	Deviation in Period 2005–2010 (%)	Deviation in Period 2010–2015 (%)	Deviation in Period 2005–2020 (%)	Deviation in Period 2005–2010 (%)	Deviation in Period 2010–2015 (%)	Deviation in Period 2005–2020 (%)
Residential area	0.62	0.43	−0.17	−0.07	0.19	0.30
Roads and rail networks and associated land	0.01	-	−0.04	0.37	−0.61	0.72
Service and industry area	0.02	0.01	−0.01	0.06	0.30	0.04
Ports and airports	-	-	-	-	-	-
Mining area, construction area	0.04	−0.05	0.01	0.01	0.09	0.01
Parks, and sport and leisure area	0.02	0.01	−0.04	−0.04	0.01	0.04
Meadows and pastures	0.12	−1.61	1.04	0.17	0.17	−0.99
Arable land	−1.20	−0.08	0.53	−0.34	−0.60	−0.74
Orchards, vineyards, and plantations	-	-	-	-	-	0.03
Other non-categorized areas	-	-	-	0.02	0.01	-
Forest area	0.46	0.34	−1.05	0.32	0.49	0.27
Scrub and/or herbaceous vegetation associations	−0.06	0.74	−0.30	0.07	0.45	0.20
Bare land (areas with little vegetation)	-	0.04	0.06	0.06	−0.50	0.10
Wetland	−0.01	-	0.16	-	-	-
Water area	−0.02	0.15	−0.18	−0.01	0.01	0.01
Landscape change index	1.28	1.73	1.80	0.78	1.74	1.74

**Table 3:** Area of each character of landscape changes in different time periods in the Mysłakowice commune and the city of Jelenia Góra.

Character of Landscape Changes	Mysłakowice			Jelenia Góra		
	Occurrence in Period 2005–2010 (ha)	Occurrence in Period 2010–2015 (ha)	Occurrence in Period 2005–2020 (ha)	Occurrence in Period 2005–2010 (ha)	Occurrence in Period 2010–2015 (ha)	Occurrence in Period 2005–2020 (ha)
Urbanization	23.25	36.71	17.82	9.41	25.01	25.56
Industrialization	0.88	0.82	-	5.12	30.48	0.68
Development of transportation areas	0.06	-	0.15	6.45	3.52	23.10
Development of recreational areas	0.43	2.20	-	-	0.20	0.56
Intensification of agriculture	24.07	6.11	5.64	8.09	15.35	47.62
Extensification of agriculture	10.23	2.53	132.72	24.02	57.75	240.59
Set-aside land	-	3.53	-	2.25	0.92	-
Afforestation	5.15	23.41	0.89	1.31	57.59	-
Deforestation	-	0.63	5.24	9.03	35.18	0.30
Natural succession	-	38.71	0.21	16.85	14.72	15.03
Water resource management	17.07	24.66	19.42	0.06	0.80	0.10
Wetlands	2.18	-	0.88	-	-	-

**Table 4:** Demographic characteristics of the survey participants.

Variable	Mysłakowice		Jelenia Góra	
	No	%	No	%
	Duration of residence			
Less than 5 years	0	0	0	0
5–10 years	1	3.1	0	0
11–15 years	3	9.4	9	20.5
16–20 years	10	31.3	9	20.5
More than 20 years	18	56.3	26	59.1
	Age			
Below 20	0	0	0	0
21–30	1	3.1	9	20.5
31–40	10	31.3	15	34.1
41–50	14	43.8	16	36.3
51–60	6	18.8	3	6.8
Over 60	1	3.1	1	2.3
	Education			
Primary	0	0	0	0
Professional	2	6.3	1	2.3
Secondary	11	34.4	12	27.3
Higher	19	59.4	31	70.5
	Social status			
Student	0	0	5	11.4
Employed	29	90.6	37	84.1
Unemployed	0	0	0	0
Pensioner	3	9.4	2	4.5
	Assessment of the level of landscape change			
Low	1	3.1	0	0
Medium	9	28.1	19	43.2
High	22	68.8	25	56.8

## Data Availability

In this research, we used archival spatial orthophoto maps for the years 2005, 2010, 2015 and 2020 from the Central Office of Cartography and Geodesy, accessed via the website www.geoportal.gov.pl (accessed on 1 December 2021). All other data are stored in the archive of the Institute of Spatial Management, Wroclaw University of Environmental and Life Sciences.

## Supplementary Materials

The following supporting information can be downloaded at: www.mdpi.com/article/10.3390/ijerph191610462/s1, Supplementary Materials File S1: The questionnaire for the municipality of Jelenia Góra; Supplementary Materials File S2: The questionnaire for the municipality of Mysłakowice.
