# Supplementary material for: Identification and Assessment of the Driving Forces behind Changes in the Foothill Landscape: Case Studies of the Mysłakowice and Jelenia Góra Communities in Poland
Source: Int J Environ Res Public Health. 2022 Aug 22;19(16):10462. doi: 10.3390/ijerph191610462 (PMC9408535; doi:10.3390/ijerph191610462)
Supplement: Supplementary file 1 [file ijerph-19-10462-s001.zip › ijerph-1832271-Supplementary File S1.pdf]

International Journal of
Environmental Research and Public Health

MDPI

Supplementary File S1

The questionnaire for the municipality of Jelenia Góra

1. **What neighborhood in Jelenia Góra do you live in? (open question)**
2. **How long have you been a resident of the city of Jelenia Góra?**
   - less than 5 years
   - 5-10 years
   - 11-15 years
   - 16-20 years
   - more than 20 years
3. **Have you noticed any changes in the landscape between 2005 and 2020? (If the answer is no please go to question 5)**
   - no
   - low
   - medium
   - high
4. **Could you identify the 3 most important landscape transformations in the city between 2005 and 2010, along with whether the change was positive or negative? (open question)**
5. **In your opinion, which of the listed time periods was characterized by the greatest intensity of change?**
   - 2005-2010
   - 2010-2015
   - 2015-2020
6. **In which part of the city do you think changes occur most frequently? (open question)**
7. **What areas have been the most prone to change in your opinion? (any number of answers)**
   - residential area
   - roads and rail networks and associated land
   - service and industry area
   - mining area, construction area
   - parks, sport and leisure area
   - meadows and pastures
   - arable land
   - orchards, vineyards and plantations
   - forest area
   - scrub and/or herbaceous vegetation associations
   - bare land (areas with little vegetation)
   - water area or wetland
8. **What areas do you think has come the most in the city over the years 2005-2020? (any number of answers)**
   - residential area
   - roads and rail networks and associated land
   - service and industry area
   - mining area, construction area
   - parks, sport and leisure area
   - meadows and pastures
   - arable land
   - orchards, vineyards and plantations
   - forest area
   - scrub and/or herbaceous vegetation associations
   - bare land (areas with little vegetation)

- water area or wetland

9. **What transformations do you think have taken place most often? (any number of answers)**
   - changes resulting from the emergence of new development
   - changes within the existing development
   - changes in types of crops
   - changes within forest areas
   - changes associated with the extraction of mineral deposits
   - emergence of new transportation areas
   - emergence of new recreational areas
   - emergence of new water reservoirs
   - natural succession in meadow areas and in areas of arable land
   - other

10. **Which of the following forces could have affected the change shown below? (any number of answers)**

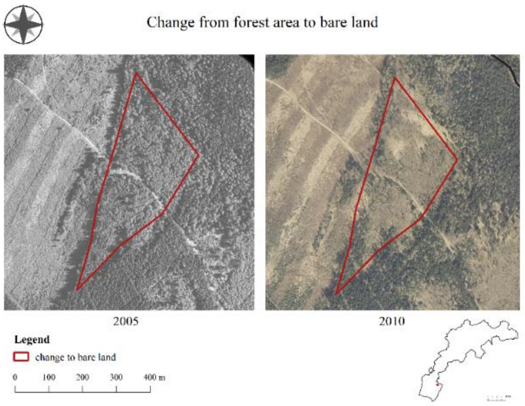

   - agricultural and foresty policy
   - nature conservation policy
   - spatial developmen policy
   - property rights
   - policy climate
   - structural change in agriculture and forestry
   - real estate market
   - prices for agricultural and forestry products
   - commercialization
   - population numbers and its structure
   - public attitudes, values and beliefs
   - individual and hosehold behavior
   - technological modernization of society
   - technological modernization in land management
   - climate
   - disturbances
   - soil characteristics
   - topography
   - extraction of deposits
   - development of areas
   - construction of water reservoirs
   - natural disaster
   - other

11. **How do you evaluate the above change?**
   - 1 - very negative impact on the landscape

- 2 - negative impact on the landscape
- 3 - neutral impact on the landscape
- 4 - positive impact on the landscape
- 5 - very positive impact on the landscape

12. Which of the following forces could have affected the change shown below? (any number of answers)

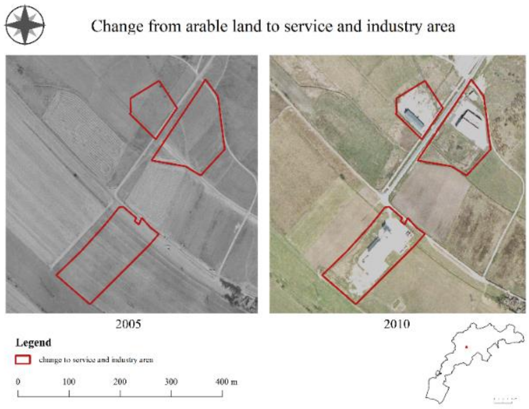

- agricultural and foresty policy
- nature conservation policy
- spatial developmen policy
- property rights
- policy climate
- structural change in agriculture and forestry
- real estate market
- prices for agricultural and forestry products
- commercialization
- population numbers and its structure
- public attitudes, values and beliefs
- individual and hosehold behavior
- technological modernization of society
- technological modernization in land management
- climate
- disturbances
- soil characteristics
- topography
- extraction of deposits
- development of areas
- construction of water reservoirs
- natural disaster
- other

13. How do you evaluate the above change?

- 1 - very negative impact on the landscape
- 2 - negative impact on the landscape
- 3 - neutral impact on the landscape
- 4 - positive impact on the landscape
- 5 - very positive impact on the landscape

14. Which of the following forces could have affected the change shown below? (any number of answers)

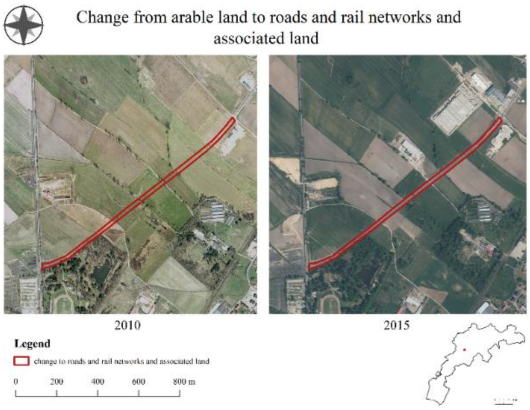

- agricultural and foresty policy
- nature conservation policy
- spatial developmen policy
- property rights
- policy climate
- structural change in agriculture and forestry
- real estate market
- prices for agricultural and forestry products
- commercialization
- population numbers and its structure
- public attitudes, values and beliefs
- individual and hosehold behavior
- technological modernization of society
- technological modernization in land management
- climate
- disturbances
- soil characteristics
- topography
- extraction of deposits
- development of areas
- construction of water reservoirs
- natural disaster
- other

15. How do you evaluate the above change?

- 1 - very negative impact on the landscape
- 2 - negative impact on the landscape
- 3 - neutral impact on the landscape
- 4 - positive impact on the landscape
- 5 - very positive impact on the landscape

16. Which of the following forces could have affected the change shown below? (any number of answers)

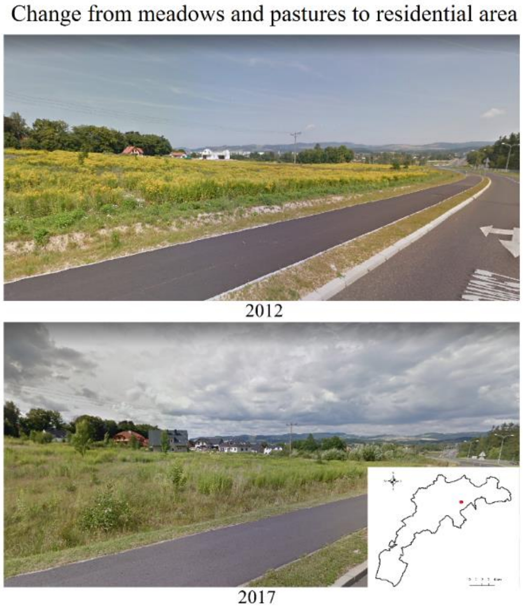

- agricultural and foresty policy
- nature conservation policy
- spatial developmen policy
- property rights
- policy climate
- structural change in agriculture and forestry
- real estate market
- prices for agricultural and forestry products
- commercialization
- population numbers and its structure
- public attitudes, values and beliefs
- individual and hosehold behavior
- technological modernization of society
- technological modernization in land management
- climate
- disturbances
- soil characteristics
- topography
- extraction of deposits
- development of areas
- construction of water reservoirs
- natural disaster
- other

17. How do you evaluate the above change?

- 1 - very negative impact on the landscape
- 2 - negative impact on the landscape
- 3 - neutral impact on the landscape
- 4 - positive impact on the landscape
- 5 - very positive impact on the landscape

18. Which of the following forces could have affected the change shown below? (any number of answers)

Change from arable land to service and industry area

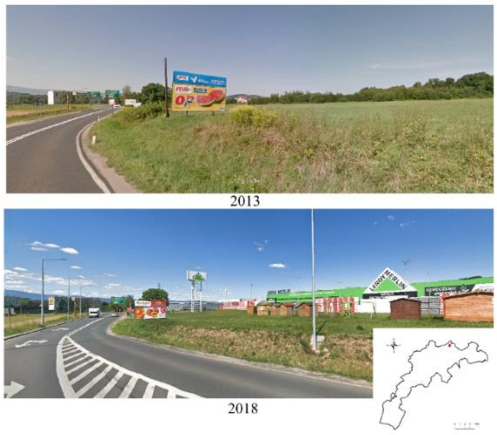

- agricultural and foresty policy
- nature conservation policy
- spatial developmen policy
- property rights
- policy climate
- structural change in agriculture and forestry
- real estate market
- prices for agricultural and forestry products
- commercialization
- population numbers and its structure
- public attitudes, values and beliefs
- individual and hosehold behavior
- technological modernization of society
- technological modernization in land management
- climate
- disturbances
- soil characteristics
- topography
- extraction of deposits
- development of areas
- construction of water reservoirs
- natural disaster
- other

19. How do you evaluate the above change?

- 1 - very negative impact on the landscape
- 2 - negative impact on the landscape
- 3 - neutral impact on the landscape
- 4 - positive impact on the landscape
- 5 - very positive impact on the landscape

20. **Which of the following forces could have affected the change shown below? (any number of answers)**

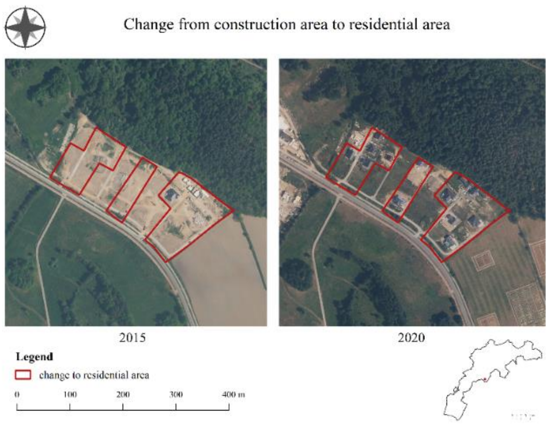

- agricultural and foresty policy
- nature conservation policy
- spatial developmen policy
- property rights
- policy climate
- structural change in agriculture and forestry
- real estate market
- prices for agricultural and forestry products
- commercialization
- population numbers and its structure
- public attitudes, values and beliefs
- individual and hosehold behavior
- technological modernization of society
- technological modernization in land management
- climate
- disturbances
- soil characteristics
- topography
- extraction of deposits
- development of areas
- construction of water reservoirs
- natural disaster
- other

21. **How do you evaluate the above change?**
- 1 - very negative impact on the landscape
- 2 - negative impact on the landscape
- 3 - neutral impact on the landscape
- 4 - positive impact on the landscape
- 5 - very positive impact on the landscape

22. **Which of the following forces could have affected the change shown below? (any number of answers)**

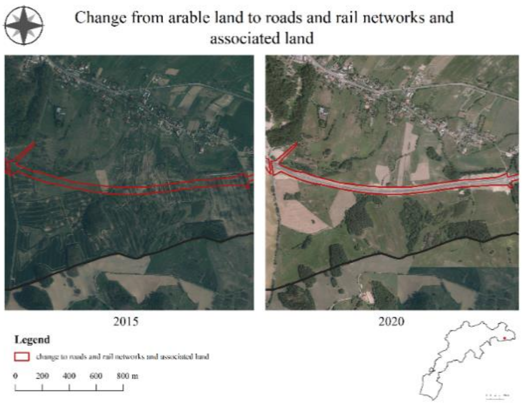

– agricultural and foresty policy
– nature conservation policy
– spatial developmen policy
– property rights
– policy climate
– structural change in agriculture and forestry
– real estate market
– prices for agricultural and forestry products
– commercialization
– population numbers and its structure
– public attitudes, values and beliefs
– individual and hosehold behavior
– technological modernization of society
– technological modernization in land management
– climate
– disturbances
– soil characteristics
– topography
– extraction of deposits
– development of areas
– construction of water reservoirs
– natural disaster
– other

23. **How do you evaluate the above change?**
   – 1 - very negative impact on the landscape
   – 2 - negative impact on the landscape
   – 3 - neutral impact on the landscape
   – 4 - positive impact on the landscape
   – 5 - very positive impact on the landscape

24. **Your own opinion on the changes in the landscape of the commune. What has most influenced the existing living conditions, etc.? (open question)**

25. **Gender**
   – male
   – female

26. **Age (open question)**

27. **Education**
    - primary
    - professional
    - secondary
    - higher
28. **Social status**
    - student
    - employed
    - unemployed
    - pensioner
